# Transformed tissue of *Dionaea muscipula* J. Ellis as a source of biologically active phenolic compounds with bactericidal properties

**DOI:** 10.1007/s00253-021-11101-8

**Published:** 2021-01-15

**Authors:** Wojciech Makowski, Aleksandra Królicka, Anna Nowicka, Jana Zwyrtková, Barbara Tokarz, Ales Pecinka, Rafał Banasiuk, Krzysztof Michał Tokarz

**Affiliations:** 1 Department of Botany, Physiology and Plant Protection, Faculty of Biotechnology and Horticulture, University of Agriculture in Krakow, Krakow, Poland; 2 Intercollegiate Faculty of Biotechnology UG and MUG, Laboratory of Biologically Active Compounds, University of Gdansk, Gdansk, Poland; 3 Institute of Experimental Botany, Czech Acad Sci, Centre of the Region Haná for Biotechnological and Agricultural Research, Olomouc, Czech Republic; 4 The Franciszek Górski Institute of Plant Physiology, The Polish Academy of Sciences, Krakow, Poland; 5 Institute of Biotechnology and Molecular Medicine, Gdansk, Poland

**Keywords:** Phenolic acids, Plumbagin, *Rhizobium rhizogenes*, Teratomas, Venus flytrap

## Abstract

**Abstract:**

The Venus flytrap (*Dionaea muscipula* J. Ellis) is a carnivorous plant able to synthesize large amounts of phenolic compounds, such as phenylpropanoids, flavonoids, phenolic acids, and 1,4-naphtoquinones. In this study, the first genetic transformation of *D. muscipula* tissues is presented. Two wild-type *Rhizobium rhizogenes* strains (LBA 9402 and ATCC 15834) were suitable vector organisms in the transformation process. Transformation led to the formation of teratoma (transformed shoot) cultures with the bacterial *rol*B gene incorporated into the plant genome in a single copy. Using high-pressure liquid chromatography, we demonstrated that transgenic plants were characterized by an increased quantity of phenolic compounds, including 1,4-naphtoquinone derivative, plumbagin (up to 106.63 mg × g^−1^ DW), and phenolic acids (including salicylic, caffeic, and ellagic acid), in comparison to non-transformed plants. Moreover, *Rhizobium*-mediated transformation highly increased the bactericidal properties of teratoma-derived extracts. The antibacterial properties of transformed plants were increased up to 33% against *Staphylococcus aureus*, *Enterococcus faecalis*, and *Escherichia coli* and up to 7% against *Pseudomonas aeruginosa*. For the first time, we prove the possibility of *D. muscipula* transformation. Moreover, we propose that transformation may be a valuable tool for enhancing secondary metabolite production in *D. muscipula* tissue and to increase bactericidal properties against human antibiotic-resistant bacteria.

**Key points:**

• *Rhizobium-mediated transformation created Dionaea muscipula teratomas*.

• *Transformed plants had highly increased synthesis of phenolic compounds*.

• *The MBC value was connected with plumbagin and phenolic acid concentrations*.

## Introduction

*Rhizobium rhizogenes* (former: *Agrobacterium rhizogenes*) is bacteria from the family Rhizobiaceae and is a “natural genetic engineer” because of the ability to transfer T-DNA (transfer DNA) and incorporate bacteria-derived genes into the plant genome (Georgiev et al. 2007). The natural features of this Gram-negative soil bacterium are used in the plant biotechnology for obtaining transformed organisms with new unique properties. During T-DNA transfer to plant cells, *R. rhizogenes* pass on the set of *rol* genes occurring in Ri plasmids (root-inducing plasmids). These genes encode specific proteins responsible for control over auxin and cytokinin synthesis in plant cells (Guillon et al. 2006). Usually, the consequence of *R. rhizogenes* infection is the change in plant hormone balance and rise of the hairy root phenotype. However, depending on the plant genotype, transformed shoots (teratomas) may occur instead of hairy roots (Królicka et al. 2010). Despite the infection mechanism of plants by *R. rhizogenes* being known for a few decades, the physiological consequences of such events related to plant species need to be studied.

Because of the increasing demand for plant-derived phytochemicals and the consumption of herbal medicines (Canter et al. 2005), genetic transformation found application in the field of medicinal plants (Niazian 2019). Hairy roots or teratomas of medicinal plants characterized by a fast growth rate and biochemical stability are promising sources of plant secondary metabolites in large-scale propagation (Georgiev et al. 2007). Moreover, they can serve as a scientific model for studies on secondary metabolism engineering and the overproduction of phytochemicals of interests in plant tissue culture (Tusevski et al. 2017). To the best of our knowledge, many *Rhizobium*-mediated transformations were successfully established in medicinal plants (Królicka et al. 2001; Gangopadhyay et al. 2010; Libik-Konieczny et al. 2020), while transformation protocol for carnivorous plant *Dionaea muscipula* J. Ellis (Venus flytrap) is still missing.

Carnivorous plants belonging to the family Droseraceae have been used in natural medicine for centuries (Królicka et al. 2010). The healing properties of these plants arise from the ability to produce large amounts of phenolic compounds with strong biological activity (Gaascht et al. 2013). Extracts from carnivorous plant tissues were proven to have antibacterial (Krolicka et al. 2008; Makowski et al. 2020), antioxidative (Królicka et al. 2009; Makowski et al. 2020), antifungal (Padhye et al. 2010), and anticancer properties (Kawiak et al. 2019). Moreover, previous phytochemical studies showed that the species most abundant in phenolic derivatives in the family Droseraceae is *D. muscipula* (Gaascht et al. 2013). The major 1,4-naphtoquinone derivative in the biochemical composition of the Venus flytrap is plumbagin. Furthermore, concentrations of this metabolite in *D. muscipula* tissue are higher than in other commonly used sources of plumbagin, like *Plumbago* plants (Makowski et al. 2020).

Since plants from the family Droseraceae are endangered species and exploitation of natural habitats is impossible, in vitro propagation protocols were established (Banasiuk et al. 2012). Therefore, the implementation of biotechnological tools for the enhancement of secondary metabolite production, like elicitation (Putalun et al. 2010; Boonsnongcheepa et al. 2019) or genetic transformation became possible (Królicka et al. 2010). Nevertheless, increasing valuable phytochemicals with elicitation and obtaining transformed plants is a difficult task because of carnivorous plant biology (Blehova et al. 2015; Makowski et al. 2019).

The main goals of the presented study were (i) transformation of the medicinal plant *D. muscipula* using wild strains of *R. rhizogenes* bacteria, (ii) with simultaneously increased synthesis of phenolic compounds, in particular plumbagin, and (iii) evaluation of bactericidal properties of extracts derived from transformed plants against clinical strains of pathogenic bacteria. We hypothesized that inoculation of the Venus flytrap with *Rhizobium* bacteria would incorporate *rol* genes into plant DNA. The purpose was to establish transformed clones characterized by fast growth and high productivity of valuable secondary metabolites with strong biological activity.

## Materials and methods

### Plant materials and bacterial strains used for transformation

*D. muscipula* plants were propagated in in vitro conditions, according to Makowski et al. (2019). Whole plant tissue cultures were cultivated on ½ MS medium (Murashige and Skoog 1962) with no growth regulators, 3% sucrose, and pH 5.5 (adjusted before autoclaving), solidified with 0.75% of agar. Conditions included a temperature of 23 ± 1 °C, fluorescence light of 80 μmol × m^−2^ × s^−1^ photosynthetic photon flux density (PPFD), and a photoperiod of 16 h/8 h light/dark cycle. Plants were subcultured in 30-day intervals.

Wild *R. rhizogenes* strains, including LBA 9402 (NCPPB 1855), ATCC 15834, and A4 (ATCC 31798) were obtained from the Laboratory of Biologically Active Compounds, University of Gdansk, Poland. Bacteria were grown on yeast extract beef (YEB) agar medium with 200 μM of acetosyringone (Sigma) at 26 °C in the dark. For plant transformation, 48-h bacterial cultures were used.

### Transformation of *D. muscipula* plants

Young (4-week-old) Venus flytrap rhizomes (150 pieces) were inoculated for each *R. rhizogenes* bacteria strain. Inoculation was performed with preparation needle, according to Królicka et al. (2010). After inoculation, rhizomes were subcultured to ½ MS medium supplemented with 3% sucrose and 0.8% of agar with pH 5.5 and grown for 3 days in the dark. Next, co-cultures were transferred to ½ MS medium supplemented with antibiotics, claforan and carbenicillin (500 mg × L^−1^ each), to eliminate *R. rhizogenes.* After 4 weeks of cultivation in the dark, new transformed shoots of *D. muscipula* were excised and placed on fresh MS medium with the same antibiotic concentrations listed above and grown in the dark for 8 weeks. After 7 subcultures, axenic cultures were established from a single shoot of transgenic tissue. Next, transformed clones were subcultured on fresh MS media without antibiotic growth regulators. Transformed clones of *D. muscipula* were propagated for 8 weeks in liquid media, as described by Makowski et al. (2020). During this time, observations of plant morphology, growth rate, and preliminary screening for phenolic compound quantity in comparison to non-transformed plants were performed. Based on these observations, four transformed clones of *D. muscipula* were chosen for further analysis.

Transformed cultures were also tested for the presence of live *R. rhizogenes* found in tissue. Transformed shoots were homogenized and the obtained suspensions were plated on YEB agar medium and grown for 5 days at 26 °C in the dark.

### Molecular analysis

To confirm transformation on a molecular level, a PCR reaction for the detection of bacteria T-DNA fragments in the plant genome was performed. To estimate the number of bacterial genes copies in plant genome Southern hybridization was used. Total genomic DNA from transformed and non-transformed *D. muscipula* plants was isolated using the CTAB method by Bekesiova et al. (1999). This method yields high-quality DNA, free from secondary metabolites. As a positive control in PCR, plasmid DNA from bacteria cells was used. A culture of 24-h old *R. rhizogenes* (OD_600_ 4.0) was extracted using alkaline lysis as described by Maniatis et al. (1982). Oligonucleotide primers for the PCR detection of *rol*B (forward primer 5′-GCTCTTGCAGTGCTAGATTT-3′, reverse primer 5′-GAAGGTGCAAGCTACCTCTC-3′), *rol*C (forward primer 5′-CTCCTGACATCAAACTCGTC-3′, reverse primer 5′-TGCTTCGAGTTATGGGTACA-3′), and *vir*G (forward primer 5′-ACTGAATATCAGGCAACGCC-3′, reverse primer 5′-GCGTCAAAGAAATAGCCAGC-3′) were used (Królicka et al. 2010). PCR was performed in three biological replicates for each examined transformed clone and non-transformed plant.

Southern hybridization was performed to evaluate the number of *rol*B and *rol*C gene copies incorporated in the plant genome. The probes for hybridization specific for *rol*B and *rol*C genes were prepared from Ri Plasmid and directly labeled using PCR with biotin-dUTP. Plant genomic DNA was isolated as described in the previous section. Two micrograms DNA was digested overnight at 37 °C with 1 unit of *Bam*HI enzyme (Thermo Fisher Scientific, Waltham, MA, USA). Subsequently, the samples were electrophoretically separated overnight on 1.2% 2-amino-2-(hydroxymethyl)-1,3-propanediol (TRIS)–borate–EDTA agarose gels, depurinated, denatured, and neutralized as described (Nowicka et al. 2020). Blotting was performed for 7 h on Hybond™ N+ nylon membrane (GE Healthcare, Chicago, IL, USA) with 20× SSC, washed in 2× SSC, dried, and crosslinked using UV Stratalinker (Agilent, Santa Clara, CA, USA). Pre-hybridization, overnight hybridization, and post-hybridization washes were performed as described in (Nowicka et al. 2020).

Hybridization points were detected using the Chemiluminescent Nucleic Acid Detection Module Kit (Thermo Fisher Scientific, Waltham, MA, USA). To visualize, resulted signals were used Medical X-Ray Film Blue (Agfa Healthcare, Mortsel, Belgium).

### Determination of biometric parameters

To estimate the growth of examined plants, transformed clones and non-transformed plants were harvested and weighed immediately. A growth index (GI) was calculated according to the formula: GI [%] = (FW_2_ − FW_1_)/FW_2_ × 100, where FW_1_ was the fresh weight of plants at the beginning of the experiment and FW_2_ was a final fresh weight. To determine dry weight (DW) accumulation, plants were freeze-dried for 72 h and weighed. DW content in plant tissue was calculated according to the formula: DW [%] = DW_2_ × 100/FW_2_, where DW_2_ was dry weight after freeze-drying. Freeze-dried plant tissue was homogenized and stored at − 20 °C for further analysis.

### Biochemical analysis

Spectrophotometric and HPLC analysis were performed to estimate level of phenolic compounds in transformed and non-transformed plants. Total phenolic content was estimated according to the method by Swain and Hillis (1959) with Folin-Ciocalteu’s reagent, after modifications (Tokarz et al. 2018). Freeze-dried tissue (10 mg) was extracted in 80% MeOH at 4 °C. Samples were centrifuged for 15 min (25,155 g, 4 °C). One milliliter of diluted extract was mixed with 0.2 mL Folin’s reagent (Sigma-Aldrich Chemie, GmBH, Steinheim, Germany) and 1.6 mL 5% Na_2_CO_3_ and incubated for 20 min at 40 °C. The absorbance of the mixture was measured at 740 nm using a Double Beam spectrophotometer U-2900 (Hitachi High-Technologies Corporation, Japan). Chlorogenic acid (Sigma-Aldrich Chemie, GmBH, Steinheim, Germany) was used as a reference standard. Results were expressed as milligram of chlorogenic acid equivalents per 1 g of DW. Analyses were performed in five biological replicates.

To determine the accumulation of phenylpropanoids, flavonoids, and anthocyanins in plant tissue, a method described by Fukumoto and Mazza (2000) was used with modifications (Tokarz et al. 2020). Tissue was extracted as described in the method for total phenolic content estimation. The supernatant (0.25 mL) was mixed with 0.25 mL 0.1% HCl in 96% EtOH and 4.55 mL 2% HCl in H_2_O. Test tubes with mixtures were incubated for 20 min in the dark. The absorbance of samples was measured at wavelengths of 320, 360, and 520 nm. Contents of phenylpropanoids, flavonoids, and anthocyanins were calculated using calibration curves made for caffeic acid, quercetin, and cyanidin (Sigma-Aldrich Chemie, GmBH, Steinheim, Germany), respectively. The results were expressed as milligrams of standard equivalents per 1 g of DW. Analyses were performed in five replicates.

To estimate plumbagin content, 10 mg of freeze-dried plant tissue was extracted in 0.6 mL of MQ water and 0.6 mL of tetrahydrofuran (THF) (Tokarz et al. 2018). For analysis of caffeic acid, hyperoside, ellagic acid, salicylic acid, myricetin, and quercetin dry tissue (20 mg) was extracted in 2 mL of 100% methanol and sonicated for 30 min (Makowski et al. 2020). Samples were centrifuged (15 min, 25,155 g, 4 °C) and filtered. Chromatographic separation was performed according to Makowski et al. (2020) using Dionex UltiMate 3000 HPLC system equipped with a quaternary pump, autosampler, column oven, and UV detector. For the stationary phase, Agilent Zorbax SB-Phenyl (4.6 × 150 mm, 3.5 μm) was used. The flow rate was 1 mL × min^−1^. The sample injection volume was 10 μL. The mobile phase for the analysis consisted of 0.1% (v/v) trifluoroacetic acid in acetonitrile as eluent A and 0.1% (v/v) trifluoroacetic acid in water as eluent B. The separation gradient was 0 min (10% A) → 5 min (10% A) → 12 min (90% A) → 20 min (90% A), followed by 10-min column regeneration. Chromatographic separations were carried out at 25 °C. Compounds present in examined plant tissues (plumbagin, hyperoxide, ellagic acid, myricetin, quercetin, salicylic acid, and caffeic acid) were used as standards to determine extract composition. A three-level standard curve was used for determining the concentration of the compounds 4-point. Monitoring was performed at 254 nm. Each analysis was performed in three biological replicates.

### Productivity of phenolic derivatives

The productivity (*P*) of each phenolic compound was calculated according to the formula: *P* [mg of phenolic compound/8 weeks/flask] = *A* × *B*, where *A* was the content of the phenolic compound in plant tissue after 8 weeks of growth and *B* was the fold of weight gain of one tissue culture (one flask).

### Antibacterial activity of plant-derived extracts

To evaluate the bactericidal properties of examined plants, minimal inhibition concentrations (MIC) and minimal bactericidal concentrations (MBC) methods were used (Królicka et al. 2009). MIC and MBC were evaluated against antibiotic-resistant, human-pathogenic bacteria: *Staphylococcus aureus* ATCC 25923, *Enterococcus faecalis* ATCC 19433, *Pseudomonas aeruginosa* ATCC 27853, and *Escherichia coli* ATCC 25922, obtained from the IFB UG & MUG Poland. The bacteria were cultivated on BHI medium (overnight, 37 °C). Freeze-dried plant tissue (100 mg) was extracted in THF (Makowski et al. 2020). Extracts were evaporated and resuspended in methanol before application into wells of the 96-well plate. To remove toxic methanol, extracts were evaporated in the wells. The residues were suspended in 100 μL of liquid BHI media, and bacterial suspensions (10 μL, 10^5^ CFU × mL^−1^) in liquid media were added to wells. Plates were incubated overnight. The MIC value was defined as the lowest concentration of applied extract that inhibits bacteria growth in the well. To establish the MBC value, 100 μL from each well that showed no visible growth of bacteria were plated out on a BHI agar plate for 24 h of incubation at 37 °C. The MBC was defined as the lowest concentration of the extract that reduced the inoculum by 99.9% within 24 h.

### Statistical analyses

One-way analysis of variance (ANOVA) was used to determine significant differences between means (Tukey test at *p* < 0.05 level). STATISTICA 12.0 (StatSoft Inc., Tulsa, OK, USA) was used to carry out statistical analyses.

## Results

### Obtaining transformed plants after inoculation with bacteria and molecular analysis

*D. muscipula* tissue was inoculated with three different strains of wild-type *R. rhizogenes*: LBA 9402, ATCC 15834, and A4. Only inoculation with LBA 9402 and ATCC 15834 resulted in the appearance of presumably transformed shoots (teratomas). Hairy root production was not observed in teratomas tissue culture. Teratomas appearing efficiency was 14% and 16% for *R. rhizogenes* LBA 9402 and ATCC 15834, respectively. Based on preliminary results from growth observations and screening for phenolic compound accumulation in teratomas tissue compared to non-transformed plants (NT plants), four transformed clones of the Venus flytrap were taken for further analysis: clones P, K, L, and E. Clones P and K were obtained after inoculation with *R. rhizogenes* LBA 9402, while L and E with ATCC 15834.

**Fig. 1:**
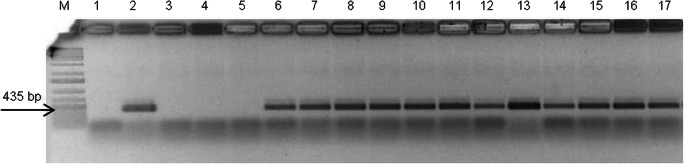
PCR analysis of non-transformed tissue of *Dionaea muscipula* (lanes 3–5) and transformed clones of *Dionaea muscipula*: clone P (lanes 6–8), clone K (lanes 9–11), clone L (lanes 12–14), and clone E (lanes 15–17). Lane 1: negative control, lane 2: positive control (*Rhizobium rhizogenes* ATCC 15834 plasmidic DNA). GeneRuler™ 100 bp DNA ladder (lane M). Bands show amplified fragments of the *rol*B gene

For molecular analysis of the incorporation of bacterial T-DNA in the plant genome, PCR detection of *rol*B and *rol*C genes was performed. Presence of the *rol*B gene was confirmed for each putatively transformed *D. muscipula* clone (P, K, L, and E), in each of three biological repetitions (samples from three independent tissue cultures), while the *rol*B gene was not detected in NT plants (Fig. 1). The presence of the *rol*C gene in transformed clones was not detected. For confirmation of *R. rhizogenes* elimination from tissue cultures of transformed plants, PCR for the *vir*G gene was performed. This gene was present in the Ri plasmid but beyond the transferred T-DNA and was not incorporated into the plant genome. *Vir*G was not found in transformed tissue cultures.

**Fig. 2:**
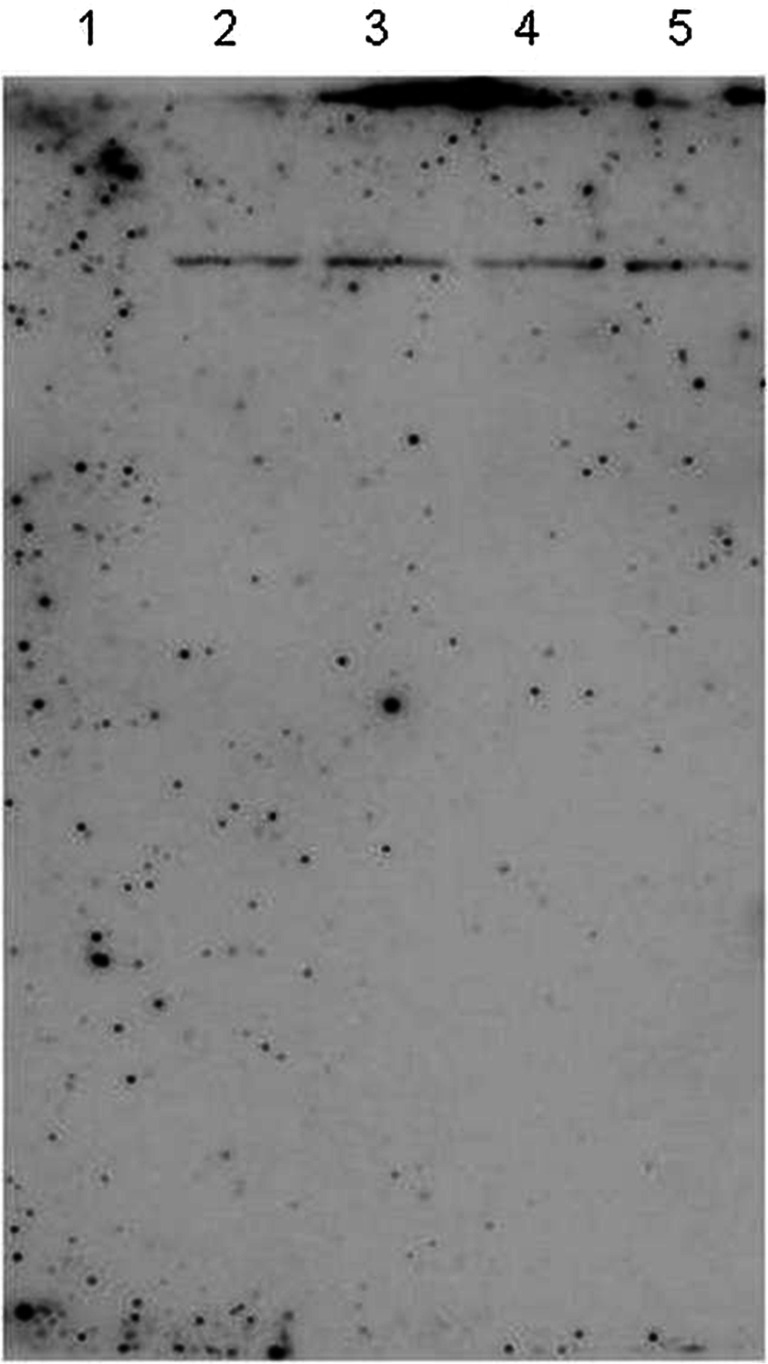
Southern hybridization with the *rol*B probe: non-transformed tissue of *Dionaea muscipula* (lane 1) and transformed clones of *Dionaea muscipula*: clone P (lane 2), clone K (lane 3), clone L (lane 4), and clone E (lane 5). The number of bands in each lane show the number of *rol*B gene copies

To estimate the number of *rol*B and *rol*C gene copies incorporated to the *D. muscipula* genome, Southern hybridization was performed. The obtained signals showed that each transformed clone of the Venus flytrap had a single copy of the searched *rol*B gene, visible as a single band in lanes 2, 3, 4, and 5 (Fig. 2), whereas the signals for *rol*C gene were not detected.

### Growth index, dry weight accumulation, and morphology of transformed clones

**Fig. 3:**
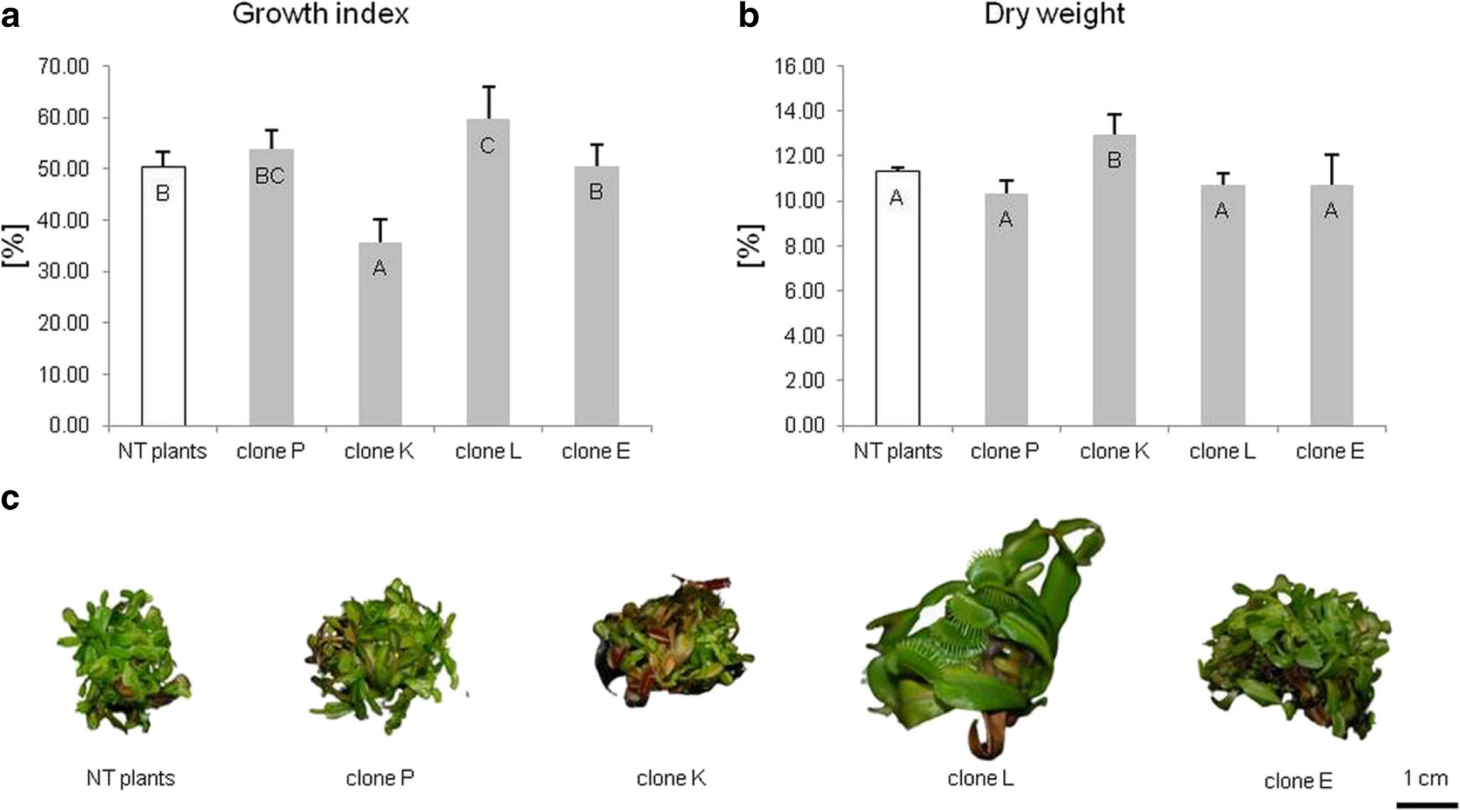
**a** Growth index (%), **b** dry weight (%), and **c** morphology of non-transformed and transformed tissue of *Dionaea muscipula* clones

After 8 weeks of cultivation of transformed clones and NT plants (control) in liquid media with rotary shaking, GI, DW content, and plant morphology were evaluated. Estimation of growth parameters showed that in comparison to NT plants, GI of clone K was significantly decreased by 29% (Fig. 3a) with simultaneously increased accumulation of DW by 15% (Fig. 3b). Clone L was characterized by 1.2-fold higher GI compared to NT plants (Fig. 3a) and changed morphology, including longer teratomas with bigger leave-traps (Fig. 3c).

### Total phenolic, phenylpropanoid, flavonoid, and anthocyanin content in transformed clones

**Fig. 4:**
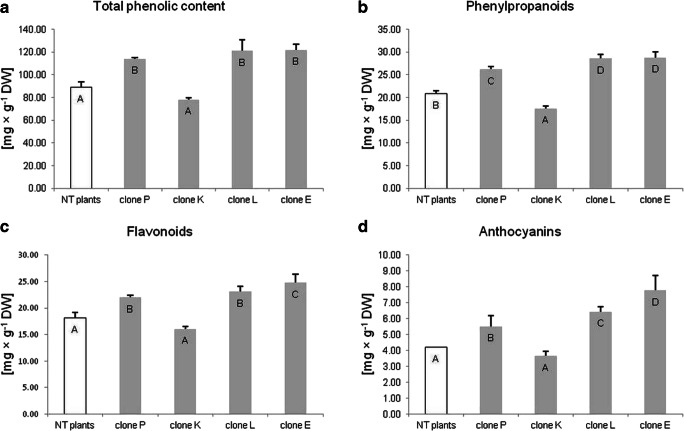
Accumulation of phenolic compounds in non-transformed and transformed tissue of *Dionaea muscipula* clones: **a** total phenolic content, **b** phenylpropanoids, **c** flavonoids, and **d** anthocyanins. Different letters indicate significant differences between means at *p* < 0.05; bar: standard deviation

Next, we examined the synthesis of phenolic compounds in transformed clones of the Venus flytrap. Using spectrophotometric methods, total phenolic content and accumulation of selected groups of phenolic derivatives were evaluated. Transgenic *D. muscipula* plants had significantly increased accumulation of total phenolic content, phenylpropanoids, flavonoids, and anthocyanins in clone P, L, and E compared to NT plants (Fig. 4). Interestingly, clone K synthesized significantly less phenolic compounds than control plants. Clone L was transformed with *R. rhizogenes* ATCC 15834 and accumulated significantly more phenylpropanoids and anthocyanins than those transformed with *R. rhizogenes* LBA 9402 (Fig. 4b and d).

### Accumulation and productivity of phenolic compound derivatives in transgenic clones

To study how the transformation of *D. muscipula* with wild strains of *R. rhizogenes* bacteria affected the production of selected phenolic compounds, HPLC was used. In comparison to NT plants, phenolic compound metabolism in transformed clones was significantly affected (Table 1). Clones P, L, and E accumulated significantly increased amounts of plumbagin, salicylic acid, and ellagic acid, while among them, clone E reached the highest level of these metabolites (1.5-, 1.9-, and 1.8-fold higher than NT plants, respectively). The level of caffeic acid was increased in clones L and E by 68 and 42%, respectively. Accumulation of hyperoside was significantly decreased in all transformed clones compared to NT plants (Table 1).

**Table 1:** Accumulation of phenolic derivatives in non-transformed and transformed tissue of *Dionaea muscipula* clones. Different letters indicate significant differences between means at *p* < 0.05, SD - standard deviation

Phenolic derivatives		NT plants	Clone P	Clone K	Clone L	Clone E
Plumbagine	mg × g^−1^ DW	69.57^A^ ± 4.15	92.40^B^ ± 4.41	74.78^A^ ± 8.85	101.35^BC^ ± 1.79	106.63^C^ ± 3.47
Hyperoside		4.47^B^ ± 0.68	2.06^A^ ± 0.28	2.52^A^ ± 0.15	2.04^A^ ± 0.36	2.33^A^ ± 0.45
Myricetin		3.17^A^ ± 0.25	3.48^A^ ± 0.05	3.37^A^ ± 0.26	3.21^A^ ± 0.20	3.67^A^ ± 0.13
Quercetin		2.21^AB^ ± 0.13	2.48^B^ ± 0.02	1.64^A^ ± 0.48	2.22^AB^ ± 0.13	2.59^B^ ± 0.08
Caffeic acid		1.16^A^ ± 0.05	1.47^AB^ ± 0.15	1.34^AB^ ± 0.15	1.96^C^ ± 0.24	1.65^BC^ ± 0.11
Salicylic acid		340.83^B^ ± 22.24	600.66^D^ ± 12.02	281.04^A^ ± 15.02	540.47^C^ ± 27.10	649.93^D^ ± 25.67
Ellagic acid		8.99^A^ ± 0.39	14.43^B^ ± 0.21	9.17^A^ ± 0.86	15.06^B^ ± 1.42	16.51^B^ ± 0.64

The productivity of phenolic compounds in obtained transgenic clones was calculated. Presented results showed that clone L was characterized by the best productivity of plumbagin, myricetin, caffeic acid, and ellagic acid, which increased 1.7-, 1.2-, 2.0-, and 2.0-fold, respectively. Clones P, L, and E had increased productivity of plumbagin, salicylic acid, and ellagic acid in comparison to NT plants, respectively. Decreased productivity of hyperoside was observed in all examined plants when compared to the control. Moreover, clone K was characterized by decreased productivity of myricetin, quercetin, and salicylic acid (Table 2).

**Table 2:** The productivity of phenolic derivatives in non-transformed and transformed tissue of *Dionaea muscipula* clones. Different letters indicate significant differences between means at *p* < 0.05, *SD* standard deviation

Phenolic compounds		NT plants	Clone P	Clone K	Clone L	Clone E
Plumbagine	mg × 8 weeks^−1^ × flask^−1^ ± SD	142.5^A^ ± 8.51	194.0^B^ ± 9.27	118.9^A^ ± 14.08	248.0^C^ ± 4.37	216.9^B^ ± 3.16
Hyperoside		9.2^B^ ± 1.40	4.3^A^ ± 0.58	4.0^A^ ± 0.24	5.0^A^ ± 0.88	4.7^A^ ± 0.92
Myricetin		6.5^B^ ± 0.50	7.3^BC^ ± 0.11	5.4^A^ ± 0.41	7.9^C^ ± 0.49	7.5^BC^ ± 0.26
Quercetin		4.5^B^ ± 0.27	5.2^B^ ± 0.04	2.6^A^ ± 0.76	5.4^B^ ± 0.32	5.3^B^ ± 0.17
Caffeic acid		2.4^AB^ ± 0.10	3.1^BC^ ± 0.32	2.1^A^ ± 0.24	4.8^D^ ± 0.58	3.4^C^ ± 0.23
Salicylic acid		697.9^B^ ± 45.55	1261.2^C^ ± 25.24	447.1^A^ ± 23.90	1322.3^C^ ± 52.22	1322.3^C^ ± 52.22
Ellagic acid		18.4^A^ ± 0.80	30.3^B^ ± 0.45	14.6^A^ ± 1.36	36.9^C^ ± 3.48	33.6^BC^ ± 1.31

### Bactericidal properties of extracts derived from transformed clones

This study focused on the synthesis of phenolic compounds in the transformed tissue of *D. muscipula* and their bactericidal properties. To estimate the antibacterial potential of transformed tissues, MIC and MBC were examined with four antibiotic-resistant, human-pathogenic bacteria. Extracts from clones P, L, and E had decreased MIC values for all tested pathogens. Similar to MBC, in the case of *P. aeruginosa*, only clones L and E were characterized by increased bactericidal properties. Antibacterial activity of clones P, L, and E against *S. aureus*, *E. faecalis*, and *E. coli* increased 33% compared to NT plants. Extracts from clones L and E had decreased MBC value against *P. aeruginosa* (7%) (Table 3).

**Table 3:** Minimal inhibition concentration (MIC) and minimal bactericidal concentration (MBC) of *S. aureus*, *E. faecalis*, *E. coli*, and *P. aeruginosa* after treatment with extracts from non-transformed and transformed tissue of *Dionaea muscipula* clones

	*Staphylococcus aureus* ATCC 25923		*Enterococcus faecalis* ATCC 19433		*Escherichia coli* ATCC 25922	*Pseudomonas aeruginosa* ATCC 27853		
	μg DW × mL^−1^							
	MIC	MBC	MIC	MBC	MIC	MBC	MIC	MBC
NT plants	167	500	667	1250	500	1250	1250	1250
Clone P	83	333	500	833	417	833	1000	1250
Clone K	167	500	833	1250	500	1250	1250	1250
Clone L	83	333	500	833	417	833	1000	1166
Clone E	83	333	500	833	417	833	1000	1166

## Discussion

To the best of our knowledge, five articles about genetic transformation of carnivorous plants are available (Hirsikorpi et al. 2002; Królicka et al. 2010; Blehova et al. 2015; Miguel et al. 2019; Oropeza-Aburto et al. 2020), while only one is about transformation with wild strains of *R. rhizogenes* bacteria (Królicka et al. 2010). Hirsikorpi et al. (2002) and Oropeza-Aburto et al. (2020) used *Rhizobium tumefaciens* (former: *Agrobacterium tumefaciens*) as a vector organism, while in the work of Blehova et al. (2015), *R. rhizogenes* with *green fluorescent protein* gene served for *Drosera rotundifolia* L. transformation. Furthermore, Miguel et al. (2019) explored virus-based plant transformation to create transgenic sundew and pitcher plants, for the overproduction of recombinant proteins. In this study, for the first time, the successful transformation of *D. muscipula* is presented.

It was shown by Franklin et al. (2008) on the *Hypericum perforatum* L. model that the most limiting factor in successful genetic transformation of plants using *Rhizobium* bacteria is plant’s recalcitrance. When plant cells are challenged with *Rhizobium,* the stress-induced defense response appears, based on induction of stress-involved gene expression patterns and up-regulation of enzymatic protein activity in the phenylpropanoid pathway, leading to increased synthesis of secondary metabolites (Franklin et al. 2009; Tusevski et al. 2019). Being a rich source of phenolic compounds with strong bactericidal properties, carnivorous plants from the family Droseraceae are hard to transform (Blehova et al. 2015), while transformation of such organisms gives the possibility to study pathways involved in the synthesis of valuable secondary metabolites and/or overproducing phytochemicals of interest (Gandhi et al. 2015). Moreover, effective transformation protocols for the Venus flytrap seem to be important tools in the field of physiological, ecological, and evolutionary research (Blehova et al. 2015).

During *Rhizobium-*mediated transformation, there are a few factors that affect the effectiveness of gene transfer from bacteria to the plant genome. One of them is the explant type (Alok et al. 2016; Hou et al. 2016). In our study, we chose rhizomes of the Venus flytrap as explants for transformation due to a lower concentration of secondary metabolites than that in leaves. The other crucial factor is the selection of the *Rhizobium* strain. Królicka et al. (2010) showed that during the transformation of *Drosera capensis* var. *alba, R. rhizogenes* ATCC 15834 strain was effective, while LBA 9402 and A4 were not conducive to transformation. Secondary metabolites contained in the leaves of medicinal plants can inhibit the growth of bacteria and decrease transformation efficiency (Królicka et al. 2010; Blehova et al. 2015). Additionally, Thiruvengadam et al. (2014a) observed differences in various *R. rhizogenes* strain effectiveness in the establishment of hairy root cultures of *Momordica charantia*. Wang et al. (2006) showed that induction of hairy roots in *Echinacea purpurea* was possible with *R. rhizogenes* A4, R1601, and R1000 strains but the performance of each strain was dependent on the type of plant explant. In contrast, the transformation rate of *Origanum vulgare* was similar for strains 15,834 and K599, while the type of medium affected hairy root appearance (Habibi et al. 2016). In our study, successful transformation of *D. muscipula* was possible with *R. rhizogenes* LBA 9402 and ATCC 15834, while the A4 strain did not caused T-DNA incorporation into plant genomic DNA. This can be the evidence that LBA 9402 and ATCC 15834 strains are less sensitive to secondary metabolites accumulated in Venus flytrap tissue, which are synthesized as defense compounds.

The presented results demonstrate that independent of the bacterial strain, obtained transformed clones of *D. muscipula* are characterized by the presence of only the *rol*B gene in plant DNA. Moreover, it was found that each clone (P, K, L, and E) had a single copy of the *rol*B gene combined in the plant genome. This result is in agreement with the findings of Królicka et al. (2010), where teratomas of transformed sundew had a single copy of the *rol*B gene. Similarly, in the research of Gangopadhyay et al. (2011), after the transformation of the medicinal plant *Plumbago indica*, obtained hairy root clones were characterized by a single copy of the *rol*B gene, which was confirmed with Southern hybridization. The type and copy number of *rol* genes (A, B, C, or D) transferred from bacteria to the plant genome during T-DNA delivery and combining, takes place accidentally, despite having a direct impact on transformed organism morphology and physiology (Ghimire et al. 2019; Ansari et al. 2019). Transforming a plant with *R. rhizogenes* usually results in hairy root culture creation but sometimes transformed cells can directly regenerate into whole plants (Blehova et al. 2015). *Rol* genes (the root loci) are essential for hairy root creation, while the phenotype of transformed plants depends on the number of *rol* genes incorporated in plant DNA and their expression pattern (Vinterhalter et al. 2015). High expression of the *rol* gene family particularly stimulates hairy root creation and elongation (Tusevski et al. 2019). The transformation of *D. muscipula* did not affect hairy root formation. These findings can result from the incorporation of only a single copy of the *rol*B gene in each clone (P, K, L, and E) and can be evidence for low expression patterns of the *rol*B gene in *D. muscipula*. Conversely, the *rol*B gene can be a suppressor of cell division and growth (Bulgakov 2008). Królicka et al. (2010) and Blehova et al. (2015) also observed direct organogenesis into whole plants (creation of teratomas) after the transformation of plants from the family Droseraceae with *R. rhizogenes.* Habibi et al. (2016) showed that the hairy root culture of the medicinal plant *O. vulgare* could regenerate into whole plants with a callus phase intervening between them. Transformed cells differentiated into whole plants can conduct changes in leaf and shoot morphology, which is called “hairy root syndrome”. Clone L was obtained after *D. muscipula* transformation and characterized by longer leaves and bigger leaf-traps than NT plants and other transformed clones, which could be the consequence of transformation.

Plant transformation with wild strains of the *R. rhizogenes* bacteria is a valuable biotechnology tool, allowing the creation of fast-growing, genetically stable organisms, with a high content of secondary metabolites (Gandhi et al. 2015). Since fast biomass accumulation in plant tissue cultures is one of the most important features in the industry field, research has been conducted to determine the growth parameters of hairy root cultures obtained after *R. rhizogenes* inoculation (Georgiev et al. 2007). Tusevski et al. (2019) showed great variation in the growth of *H. perforatum* hairy roots, although each clone was obtained using the same bacterial strain. Similar variability of growth parameters was observed by Tusevski et al. (2017). Additionally, hairy root lines of *Rehmannia elata* showed large differences in fresh weight growth rate and DW accumulation (Piątczak et al. 2019). *Polygonum multiflorum* Thunb. and *M. charantia* hairy roots reached nearly a 10-fold increase in the growth of fresh weight after 20 days (Thiruvengadam et al. 2014a, b). Binoy et al. (2016) reported a 12-fold increase in *Plumbago rosea* hairy roots. Nevertheless, little is known about the growth of teratomas. Królicka et al. (2010) demonstrated that teratomas of *D. capensis* had a three times higher growth index than non-transformed plants. In this study, for the first time, growth parameters of transformed *D. muscipula* plants were evaluated. The obtained results showed that clone L had increased GI in comparison to NT plants, while clone K accumulated significantly less biomass than control plants, with simultaneously increased DW content. In our study, *D. muscipula* transformed clones’ growth characteristics had no connection with the bacteria strain. Differences between the obtained clones using the same bacteria strain (LBA 9402 or ATCC 15834) may have resulted from the fact that each clone originated from different transformation events. Heterogeneity among transgenic clones could depend on physiological conditions of the transformed organism, expression of *rol* genes, and copy numbers of genes from bacterial T-DNA inserted in the plant genome (Tusevski et al. 2019).

Franklin et al. (2009) studied the basis of plant recalcitrance under co-cultivation with *Rhizobium* bacteria. Inoculation of plant tissue conducted for the up-regulation of gene expression involved in defense response consequently led to increased secondary metabolism and affected accumulation of phytochemicals (Hou et al. 2016). In contrast, little is known about the metabolism of secondary compounds after the incorporation of bacterial T-DNA in the plant genome. Tusevski et al. (2019) reported that hairy root culture had the same, or an even greater, ability to produce valuable metabolites. It is postulated that the *rol* genes family can act as an endogenous elicitor and conduct changes in the phytochemical profile of medicinal plants (Tusevski et al. 2019). From the industrial point of view, modified organisms can be a low cost, environmentally friendly source of valuable chemicals (Gandhi et al. 2015), while the productivity of secondary compounds can be greatly improved (Królicka et al. 2010). Increased production of total phenolic content, total flavonoids, myricetin, quercetin, caffeic acid, and salicylic acid was shown to be a consequence of the up-regulation of the *rol*C gene in hairy roots of *Ligularia fischeri* (Ansari et al. 2019). Hairy roots of *H. perforatum* showed greater activity of enzymes involved in the phenylpropanoid pathway and in consequence, accumulated significantly more total phenolic and flavonoid content in comparison to non-transformed roots (Tusevski et al. 2017). Ghimire et al. (2019) reported the increased synthesis of total phenolic and flavonoid content with simultaneously higher production of selected phenolic derivatives in hairy root cultures of *Aster scaber* and postulated the vital role of *rol* genes in the greater synthesis of these compounds. These results could be based on the phenomenon of turning on the transcription of defense genes by the *rol* genes family (Thiruvengadam et al. 2014b). Increased synthesis of phenolic compounds in hairy root cultures compared to non-transformed plants were also reported (Thiruvengadam et al. 2014a). Transformation of *D. muscipula* plants also affected the metabolism of phenolic compounds in obtained teratomas, although only the *rol*B gene was confirmed to be incorporated in the plant genome. Three of the four selected clones (P, L, and E) had significantly higher total phenolic, phenylpropanoid, flavonoid, and anthocyanins content, than NT plants. It may be connected with the fact that the *rol*B gene was proved as the strongest inducer of secondary metabolites in the *rol* gene family (Bulgakov 2008).

Additionally, analysis of the use of HPLC showed great variation in phenolic derivatives accumulation in transformed tissues of the Venus Flytrap. The highest plumbagin accumulation was observed in clone E after a 1.5-fold increase of this metabolite. Conversely, clone L was characterized by the greatest plumbagin productivity (1.7-fold increase compared to NT plants). Our observations agree with the results of Królicka et al. (2010), where teratomas of *D. capensis* had significantly increased accumulation and productivity of ramentaceone (1,4-naphtoquinone derivative) in comparison to non-transformed plants. Moreover, teratomas of *D. muscipula* accumulated over 20 times more plumbagin (106.63 mg × g^−1^ DW) than hairy roots of *P. indica* (4.9 mg × g^−1^ DW), as reported by Gangopadhyay et al. (2011). Furthermore, both accumulation and productivity of phenolic acids, like caffeic, salicylic, and ellagic acid, were increased as a consequence of transformation in some clones of *D. muscipula* created during the transformation process mediated by *R. rhizogenes* bacteria. These findings agree with previous findings (Ghimire et al. 2019; Ansari et al. 2019), where the production of phenolic acids in hairy roots of transformed plants was significantly increased in comparison to NT plants. In contrast to the results of Ansari et al. (2019), in clones of *D. muscipula*, metabolism of myricetin and quercetin was not affected by transformation, while the level of hyperoside in transformed plant tissue was decreased. In contrast to clones P, L, and E, synthesis of phenolic compounds in clone K was the same or decreased, compared to NT plants. Similar observations were reported by Wang et al. (2006) in the culture of hairy roots of *E. purpurea*. The expression pattern of *rol* genes in clone K is likely lower than in other obtained clones (Tusevski et al. 2017).

At the same time, medicinal plant tissue culture gives the possibility for producing valuable secondary metabolites and to quickly screen the biological properties of elite plant genotypes. Due to this, research on the healing properties of plant-derived extracts is available (Niazian 2019). Many studies were conducted to estimate the antimicrobial potential of medicinal plants because of the growing resistance of human pathogenic bacteria to available antibiotics (Krychowiak et al. 2014). In this article, we present for the first time, the bactericidal properties of transformed *D. muscipula* plants against Gram-positive and Gram-negative bacteria strains. It was reported by Krolicka et al. (2008) and Makowski et al. (2020) that extracts from carnivorous plants from the family Droseraceae are highly biologically active thanks to a high content of phenolic compounds, especially 1,4-naphtoquinone derivatives. According to this, biotechnological studies conducted for the improvement of healing properties in carnivorous plants seem to be needed. Ansari et al. (2019) showed that hairy roots of *L. fischeri* had greater antimicrobial activity than non-transformed tissue, against various clinical bacteria strains. Moreover, the bactericidal properties of hairy roots were improved for both Gram-positive and Gram-negative bacteria. More pronounced activity against pathogenic bacteria was also observed in hairy roots of *M. charantia* (Thiruvengadam et al. 2014a), *P. multiflorum* (Thiruvengadam et al. 2014b), and *H. perforatum* (Tusevski et al. 2017). This phenomenon is directly connected with the higher production of biologically active phytochemicals in this type of transformed tissue. In the presented study, we evaluated the antimicrobial potential of extracts derived from transformed clones of the Venus flytrap against two Gram-positive (*S. aureus* and *E. faecalis*) and two Gram-negative (*E. coli* and *P. aeruginosai*) bacteria. Clones P, L, and E showed improved bactericidal properties against *S. aureus*, *E. faecalis*, and *E. coli*, while only extracts from clones L and E had strong activity against *P. aeruginosa*. It may be connected to the increased level of salicylic acid in these clones. Our results agree with data presented by Wen et al. (2019), where *P. aeruginosa* was more resistant to *Orostachys cartilaginous*-derived extracts in comparison to the Gram-positive pathogen *Bacillus subtilis.* Except for plumbagin, which was proven to be biologically active and potent, phenolic acids have a crucial role in the antibacterial activity of medicinal plants. In this study, transformed clones of *D. muscipula* with increased bactericidal properties accumulated significantly more salicylic and ellagic acid. Gomes et al. (2018) showed that among the tested species, *Eucalyptus globules* had the highest antimicrobial activity, rich in ellagic acid glycoside. Clone K did not show improvement in antimicrobial potential, which agrees with results obtained from the estimation of secondary metabolite content. Interestingly, according to the obtained results from MBC tests, the Gram-positive pathogen *E. faecalis* had the same sensitivity to extracts derived from *D. muscipula* tissue as Gram-negative *E. coli.* This observation is in opposition to previous findings by Krolicka et al. (2008) and Makowski et al. (2020). Because Gram-positive bacteria do not have a lipopolysaccharide membrane surrounding the cell wall (Ansari et al. 2019) and permeability of antimicrobial compounds is increased (Tusevski et al. 2015), extracts from medicinal plants have stronger bactericidal properties against such pathogens. Nevertheless, the obtained results indicate that *D. muscipula* transformed clones had great bactericidal activity and can be used as a source of biologically active compounds in the pharmacological field.

## Data Availability

The datasets generated during and/or analyzed during the current study are available from the corresponding author on reasonable request.
